# Toward the use of diffuse reflection spectroscopy for intra-operative tissue discrimination during sarcoma surgery

**DOI:** 10.1117/1.JBO.29.2.027001

**Published:** 2024-02-15

**Authors:** Freija Geldof, Yvonne M. Schrage, Winan J. van Houdt, Henricus J. C. M. Sterenborg, Behdad Dashtbozorg, Theo J. M. Ruers

**Affiliations:** a Netherlands Cancer Institute, Image-Guided Surgery, Department of Surgery, Amsterdam, The Netherlands; b University of Twente, Faculty of Science and Technology, Enschede, The Netherlands

**Keywords:** diffuse reflectance spectroscopy, sarcoma surgery, tissue classification, margin assessment, machine learning

## Abstract

**Significance:**

Accurately distinguishing tumor tissue from normal tissue is crucial to achieve complete resections during soft tissue sarcoma (STS) surgery while preserving critical structures. Incomplete tumor resections are associated with an increased risk of local recurrence and worse patient prognosis.

**Aim:**

We evaluate the performance of diffuse reflectance spectroscopy (DRS) to distinguish tumor tissue from healthy tissue in STSs.

**Approach:**

DRS spectra were acquired from different tissue types on multiple locations in 20 freshly excised sarcoma specimens. A k-nearest neighbors classification model was trained to predict the tissue types of the measured locations, using binary and multiclass approaches.

**Results:**

Tumor tissue could be distinguished from healthy tissue with a classification accuracy of 0.90, sensitivity of 0.88, and specificity of 0.93 when well-differentiated liposarcomas were included. Excluding this subtype, the classification performance increased to an accuracy of 0.93, sensitivity of 0.94, and specificity of 0.93. The developed model showed a consistent performance over different histological subtypes and tumor locations.

**Conclusions:**

Automatic tissue discrimination using DRS enables real-time intra-operative guidance, contributing to more accurate STS resections.

## 1 Introduction

Soft tissue sarcoma (STS) is a rare form of cancer that accounts for <1% of all cancer cases worldwide.^1^ More than 50 histological subtypes have been identified arising from various mesenchymal tissue types, with the majority of cases located in the extremities, trunk, and retroperitoneum.^2^ Patients can be asymptomatic for a long time, and as a result, the tumor has usually grown significantly by the time it is diagnosed.

The primary treatment for sarcomas is surgery. Achieving a complete resection of the tumor with negative resection margins (no tumor cells at the resection edge) is crucial for patient outcomes. Positive resection margins are associated with an increased risk of local recurrence and worse prognosis.^3^^–^^5^ Unfortunately, identifying the tumor boundaries during surgery can be challenging and the removal of too much healthy tissue or surrounding critical structures may cause complications or difficulties with reconstruction. The incidence of positive resection margins varies per sarcoma location and subtype. Incomplete resection rates of 11 to 33% are reported for STS in general,^6^^–^^16^ which rises up to 47% for retroperitoneal located sarcomas.^6^^,^^7^ In addition, myxofibrosarcoma is known for its particularly high local recurrence rate, due to an infiltrative growth pattern, which makes it difficult for surgeons to identify the tumor borders.^17^^–^^20^ Therefore, additional therapies, such as (post-operative) radiotherapy or re-excisions, may be required to improve patient prognosis. Overall, high-grade sarcoma is associated with a relatively low 5-year survival rate of ∼60%.

Current margin assessment techniques for sarcoma surgery are limited. Frozen sections can be obtained during surgery for a simple histopathological examination of specific locations, but this process takes 30 to 60 min and the accuracy is not optimal, especially for adipocytic and (myo)fibroblastic sarcomas.^21^ The definitive resection margin status is determined by sectioning the complete excised specimen and applying the full-range of histopathological techniques. However, it may take up to 10 days before these results are available. This highlights an unmet need for an intra-operative tissue discrimination technique that can accurately and in real-time determine the resection margin status during sarcoma surgery, to ensure complete resections.

Optical techniques have emerged as a potentially valuable tool for tissue sensing purposes in the medical field. For STS, a couple of optical techniques have been studied in recent years, including near-infrared autofluorescence,^22^ Raman spectroscopy,^23^^,^^24^ mid-infrared spectroscopy,^25^ and indocyanine green fluorescence imaging (ICG).^26^ While some of these methods have demonstrated promising outcomes, not every approach is readily available, has a sufficient measurement volume, is offered as an automatic tumor detection model, or achieved an adequate level of accuracy for clinical application.

Diffuse reflectance spectroscopy (DRS) is another optical technique, which is non-invasive, real-time, easy to use, and does not require administration of contrast agents. It provides unique information about the tissue composition by illuminating the tissue with a broadband light source via an optical fiber, and subsequently measuring the reflectance spectrum after the light underwent multiple scattering and absorption interactions. Previous studies have successfully used DRS for tumor classification and surgical margin assessment in other cancer types, including breast,^27^^–^^29^ colorectal,^30^^–^^32^ head and neck,^33^ and lung tumors.^34^ In this study, we investigate the potential of DRS to distinguish tumor tissue from healthy tissue during sarcoma surgery. The tissue classification performance is evaluated in freshly excised sarcoma specimens from multiple tumor locations and various histological subtypes. To the best of our knowledge, this is the first study on the use of broadband DRS for margin assessment in STS surgery.

## 2 Materials and Methods

### 2.1 Diffuse Reflectance Spectroscopy System

The DRS system used in this study contains a halogen broadband light source (Avantes, AvaLight-HAL, 360 to 2500 nm) and two spectrometers. The first spectrometer covers the visible wavelength range (Avantes, AVASPEC-HS2048XL-EVO, 200 to 1160 nm) and the second spectrometer covers the near-infrared range (Avantes, AVASPECNIR256-1.7-RS, 900 to 1750 nm). The handheld probe consists of two fibers (400  μm diameter), with a source-to-detector distance of 2 mm. All components were controlled by in-house developed MATLAB software, which was also used to process the data.

### 2.2 Patient Inclusion

Measurements were performed on freshly excised specimens from patients who were diagnosed with sarcoma and underwent a surgical resection at the Antoni van Leeuwenhoek Hospital - Netherlands Cancer Institute. This *ex vivo* study has been performed under the approval of the Hospital Ethics Review Board (IRBm23-074), and all patients have given permission for the further use of their data and biological materials for scientific research.

### 2.3 Measurement Protocol

**Fig. 1:**
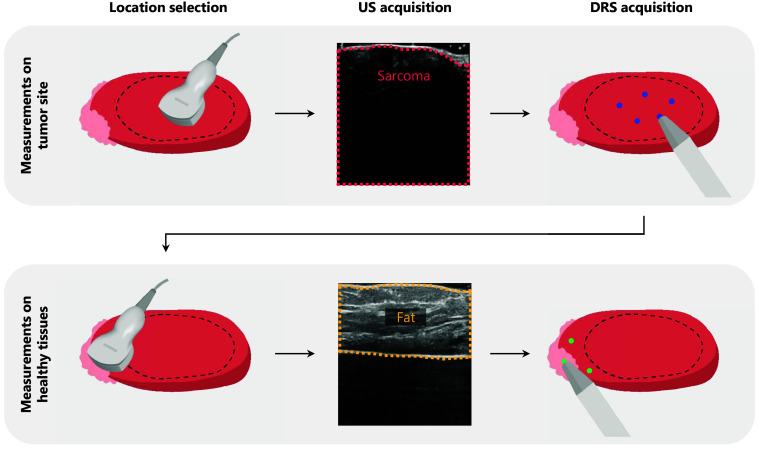
Measurement workflow. First, the location of the tumor area was determined by an expert using palpation and ultrasound imaging (shown by the dashed black line in the illustrations). Next, DRS measurements were performed at multiple locations in this area. Subsequently, surrounding locations with healthy tissue types (e.g., fat, muscle, skin) were selected by an expert using palpation and ultrasound imaging. Here, DRS measurements were performed as well. For each measured location, a corresponding US image was saved for further review by an expert. The US images in this figure show examples of sarcoma and healthy (fat) measurement locations of a leiomyosarcoma resection specimen.

The sarcoma specimens were collected from the operating room directly after surgical resection. Before performing the DRS measurements, the tumor region was identified using palpation and ultrasound imaging with a 15 MHz transducer, interpreted by an expert technical physician. Next, DRS measurements were performed successively in the tumor region and locations with different types of healthy tissue. For every measured location, a corresponding US image was saved for further review by an expert. Ground truth labels were assigned based on the expert US annotations. The number of measurement locations per tissue type depended on the tumor and specimen size. To prevent potential patient-specific biases, the measurements within each patient were acquired at locations with a minimum separation of at least 2 cm. This measurement workflow is summarized in Fig. 1, in which also two example US images are shown with their corresponding tissue labels.

### 2.4 Data Preprocessing

Several preprocessing steps were employed to optimize the raw DRS data for accurate tissue discrimination analysis. The DRS data were calibrated to correct for the sensitivity of the system and the ambient light, by measuring a white reference (Spectralon Avantes WS-2, Avantes, Apeldoorn, the Netherlands) and a dark reference (switching off the light source), as described in detail in a previous study.^35^ To create one broad spectrum per measurement location, the results from the visible and near-infrared spectrometers were stitched together in the DRS software by using the shared wavelength overlap. The spectral regions before 400 nm and after 1600 nm were removed due to low signal-to-noise ratio, leaving a wavelength range of 400 to 1600 nm for further analysis.

All DRS spectra were normalized using standard normal variate (SNV) normalization, to reduce non-tissue-related variations in the reflectance intensities.^36^ SNV normalization involves subtracting the mean of the spectrum and dividing it by the standard deviation of the spectrum.

### 2.5 Data Analysis

To predict the tissue types of the measured locations, we trained a supervised classification model. The input to the model consisted of the SNV-normalized broadband DRS spectra, obtained from the resection specimens, together with the tissue type labels. We used a weighted k-nearest neighbors (KNN) model with a 10-fold cross-validation approach. In this approach, the dataset was randomly divided into 10 subsets. Each subset was used as a validation set once while the remaining nine subset partitions were used as a training set. This process was repeated 10 times, until the performance had been assessed for all samples in the dataset.

To assess the tissue classification performance of the model, the accuracy, area under the receiver operating characteristic curve (AUC), sensitivity, specificity, and Matthews correlation coefficient (MCC) were calculated. The MCC accounts for any class imbalance in the dataset. The first four metrics have an outcome range from 0 (indicating poor prediction) to 1 (indicating perfect prediction), whereas the MCC can range from −1 to 1.

Well-differentiated liposarcomas (WDLS, also called atypical lipomatous tumors when present in the extremities) are currently being considered as borderline malignant or non-malignant. In terms of clinical relevance, this subtype might therefore be less important to detect with DRS. On the other hand, these tumors are originating from fat tissue, which may complicate the discrimination from healthy fat tissue. While similar studies^22^^,^^23^ have excluded WDLS during data analysis for this reason, in this study, we performed multiple experiments in which WDLS was considered as a separate tumor class (in a multiclass model), included in the sarcoma class (in binary model) and excluded (in both a multiclass and binary model). This approach allowed us to evaluate the performance of DRS in differentiating between WDLS and healthy fat tissue as well.

For all analyses, the results when using a binary classification model that distinguishes tumor tissue from all healthy tissues were compared to the results when using a multiclass classification model that differentiates between all different tissue types present in the resection specimens. A Chi-squared test with a significance level of 0.05 was used to assess differences in tumor classification accuracy between patients who received neoadjuvant therapy and those who did not.

## 3 Results

### 3.1 Patient and Tumor Characteristics

Freshly excised sarcoma specimens from 20 patients were included in this study. The patient and tumor characteristics are summarized in Table 1. The patient population consisted of 9 females (45%) and 11 males (55%), with a mean age of 58.4±15.4 years. A majority of the patients did not receive any neoadjuvant therapy (65%), whereas neoadjuvant chemotherapy or radiotherapy was received by 10% and 25% of the patients, respectively. Tumors were mainly located in the extremities (55%).

**Table 1:** Patient and tumor characteristics.

	Number of patients (%)
**Age** (years)	58.4±15.4
**Gender**	
Female	9 (45%)
Male	11 (55%)
**Neoadjuvant therapy**	
Chemotherapy	2 (10%)
Radiotherapy	5 (25%)
None	13 (65%)
**Tumor location**	
Extremities	11 (55%)
Retroperitoneum	6 (30%)
Trunk	3 (15%)
**Tumor type**	
Myxofibrosarcoma	4 (20%)
Well differentiated liposarcoma	3 (15%)
Leiomyosarcoma	3 (15%)
Dedifferentiated liposarcoma	2 (10%)
Myxoid liposarcoma	2 (10%)
Undifferentiated pleiomorphic sarcoma	2 (10%)
Pleiomorphic liposarcoma	1 (5%)
Undifferentiated spindel cell sarcoma	1 (5%)
Chondrosarcoma	1 (5%)
Malignant peripheral nerve sheeth tumor	1 (5%)
**Maximum tumor size** (cm)	14.5±10.0
**Resection margin** (mm)	0.73±1.48
**Resection margin status**	
R0	13 (65%)
R0 (close)	4 (20%)
R1	3 (15%)

**Fig. 2:**
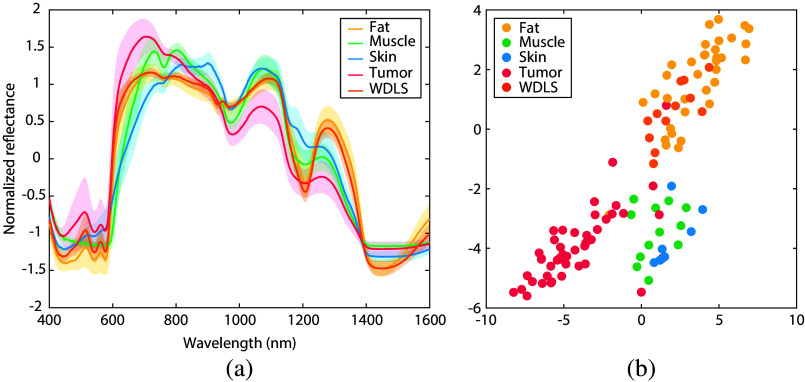
(a) Mean DRS spectra for different tissue types; fat, muscle, skin, tumor (sarcoma excluding WDLS), and WDLS. The shaded areas show the corresponding standard deviations; (b) t-SNE plot visualizing clusters in the spectral dataset after dimensionality reduction. The color of each point refers to the actual tissue type corresponding to the measured location (this information was not used by the t-SNE algorithm). WDLS, well-differentiated liposarcoma.

DRS measurements were performed on 150 locations in total (seven to eight measurements per specimen). Locations for which the spectra showed poor tissue contact (n=19) and the US images showed inconclusive labels (n=17) were removed from the dataset, to ensure that only high-quality spectra representing the tissue of interest were used for the analysis. Finally, 114 locations remained for further analysis: 55 locations on healthy tissue and 59 locations on sarcoma tissue. A breakdown of the spectral labels by tissue type and number of patients is shown in Fig. 2. The average distance to the tumor over all measured tumor locations was equal to 1.08±0.85  mm.

### 3.2 Tissue Type Classification

The average DRS spectra of the different healthy and tumorous tissue types are shown in Fig. 2(a). The spectra of sarcoma tissue differ from the spectra of healthy tissue types in multiple wavelength regions. In the wavelength ranges of 670 to 675 nm and 1085 to 1135 nm, the standard deviation of sarcoma tissue does not overlap with any other tissue type. The spectra of fat tissue and WDLS show the most similarities, with both clearly showing the reflectance dip around 1200 nm, which is characteristic of adipose tissues.

To identify any patterns or clusters with similar characteristics in our dataset, t-distributed stochastic neighbor embedding (t-SNE) was used. This nonlinear dimensionality reduction method is able to visualize the high-dimensional dataset (1200 features per DRS spectrum for 114 measured samples) in a two-dimensional space. Figure 2(b) shows the resulting data points for all measurement locations. Three distinctive clusters can be seen: one corresponding to the spectra from tumor locations, one corresponding to the spectra from muscle and skin locations, and one corresponding to the spectra from fat and WDLS locations. WDLS, however, does seem to have its own cluster within the other fat locations. The fact that t-SNE is able to separate the data into natural clusters indicates that automatic tissue classification based on DRS spectra may be feasible (Table 2).

**Table 2:** Summary of measurement locations.

Characteristic	Number of spectra	Number of patients
Fat	35	15
Muscle	13	6
Skin	7	3
Sarcoma (excluding WDLS)	47	15
WDLS	12	3

The number of spectra and patients in this table is after excluding inconclusive US images. WDLS, well-differentiated liposarcoma.

#### 3.2.1 Performance including well-differentiated liposarcomas

**Fig. 3:**
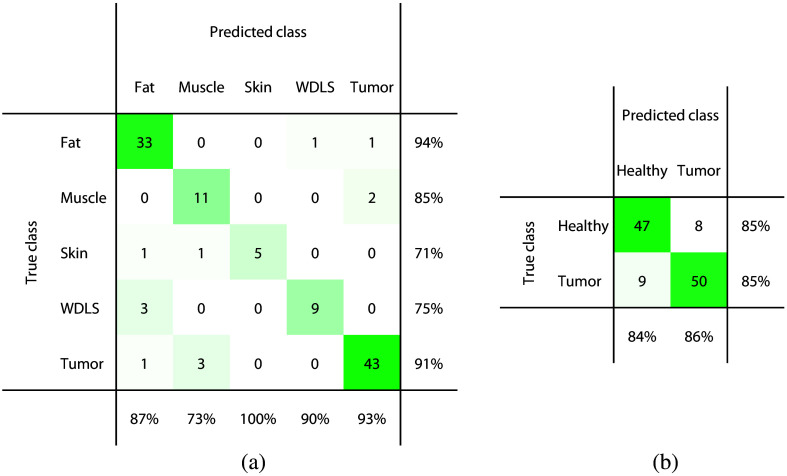
Confusion matrices showing the predicted tissue types versus the true tissue types with WDLS included in a separate class for (a) the multiclass approach and included in the tumor class for (b) the binary approach. The correctly classified measurement locations are found on the diagonal. The percentages in the last row and column specify the positive predicted values and sensitivities for the individual classes, respectively. The green color shade represents the number of locations, with darker green representing larger numbers.

The labeled DRS spectra were used to train a tissue classification model, both using a binary approach (sarcoma versus healthy) and a multiclass approach (different tissue types in five separate classes). It should be noted that in the binary approach, the healthy class consists of fat, muscle, and skin. The resulting classification performances are summarized in Table 3. Using the binary method, it was possible to discriminate sarcoma tissue from healthy tissue types with an accuracy of 0.85, sensitivity of 0.85, and specificity of 0.85. When training a multiclass kNN model, the classification performance increased to a tumor classification accuracy of 0.90, sensitivity of 0.88, and specificity of 0.93. Figure 3 shows the confusion matrices with all individual predictions, for both the binary and the multiclass classification approach.

**Table 3:** Tissue classification performance including WDLSs.

Method	Accuracy [95% CI]	Sensitivity [95% CI]	Specificity [95% CI]	AUC	MCC
Binary	0.85 [0.77; 0.91]	0.85 [0.73; 0.93]	0.85 [0.73; 0.94]	0.91	0.70
Multiclass	0.90 [0.84; 0.95]	0.88 [0.77; 0.95]	0.93 [0.82; 0.98]	0.96	0.81

**Fig. 4:**
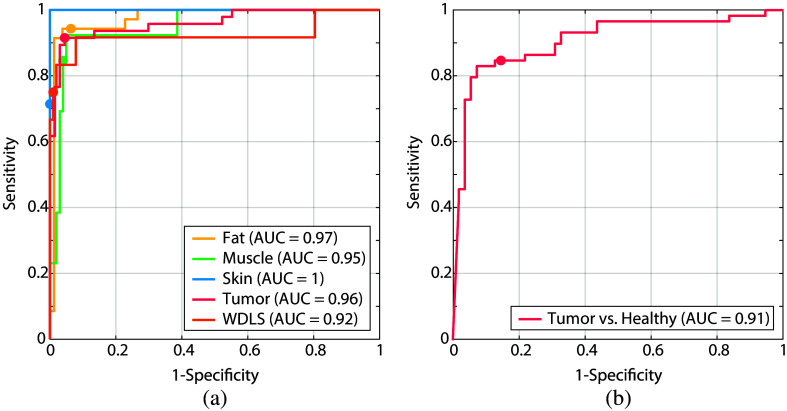
ROC curves of the tissue classification models with WDLS included in a separate class for (a) the multiclass approach and included in the tumor class for (b) the binary approach. (a) The multiclass plot shows one-versus-all curves for each class. The circular markers indicate the selected operating points of the trained model for each tissue type, for which the accuracies are reported in Table 3 and Fig. 3.

The ROC curves corresponding to the trained models are shown in Fig. 4. The AUC for tumor tissue is equal to 0.91 in case of the binary model and 0.96 for the multiclass model. In addition, healthy tissue types can be distinguished better using a multiclass approach. AUC values of 0.97, 0.95, and 1 were achieved when using separate classes of fat, muscle, and skin, compared to an AUC of 0.91 when using one general healthy class. The circular markers in the figure indicate the selected operating points of the trained model for each tissue type, for which the accuracies are reported in Table 3 and Fig. 3.

#### 3.2.2 Performance excluding well-differentiated liposarcomas

**Fig. 5:**
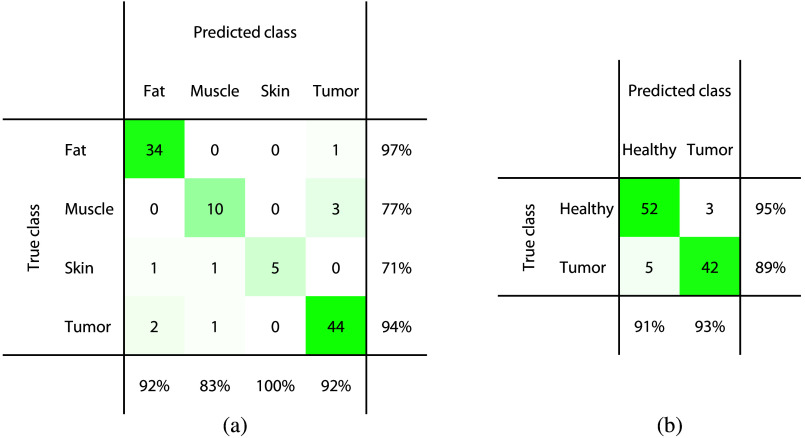
Confusion matrices showing the predicted tissue types versus the true tissue types when excluding WDLSs, for both (a) a multiclass approach and (b) binary approach. The correctly classified measurement locations are found on the diagonal. The percentages in the last row and column specify the positive predicted values and sensitivities for the individual classes, respectively. The green color shade represents the number of locations, with darker green representing larger numbers.

As described in Sec. 2.5, we also trained and evaluated a tissue classification model when excluding WDLS locations from the dataset (n=12). In this case, sarcoma tissue could be discriminated from healthy tissue types with an accuracy of 0.92, sensitivity of 0.89, and specificity of 0.95 when using a binary classification approach (see Table 4). When training a multiclass model, the performance increased to an accuracy of 0.93, sensitivity of 0.94, and specificity of 0.93. Figure 5 shows the corresponding confusion matrices with all individual predictions, for both classification approaches.

**Table 4:** Tissue classification performance excluding WDLSs.

Method	Accuracy [95% CI]	Sensitivity [95% CI]	Specificity [95% CI]	AUC	MCC
Binary	0.92 [0.85; 0.97]	0.89 [0.77; 0.96]	0.95 [0.85; 0.99]	0.94	0.84
Multiclass	0.93 [0.86; 0.97]	0.94 [0.83; 0.99]	0.93 [0.82; 0.98]	0.97	0.86

**Fig. 6:**
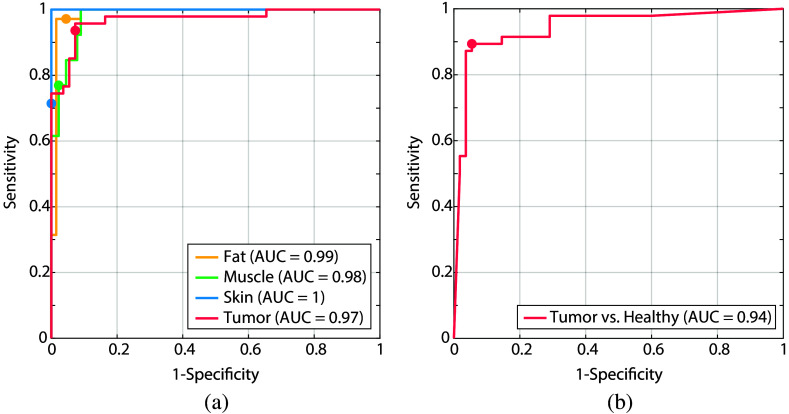
ROC curves of the tissue classification models excluding WDLSs, for both (a) a multiclass approach and (b) binary approach. (a) The multiclass plot shows one-versus-all curves for each class. The circular markers in the figure indicate the selected operating points of the trained model for each tissue type, for which the accuracies are reported in Table 4 and Fig. 5.

Figure 6 shows the ROC curves for every tissue class. The AUC for tumor tissue is equal to 0.97 in the case of a multiclass approach, compared to an AUC of 0.94 in the case of a binary approach. The AUC values for healthy tissue types are higher using the multiclass approach as well. The circular markers in the figure indicate the selected operating points of the trained model for each tissue type, for which the accuracies are reported in Table 4 and Fig. 5.

### 3.3 Classification Performance Over Different Tumor Locations and Subtypes

**Fig. 7:**
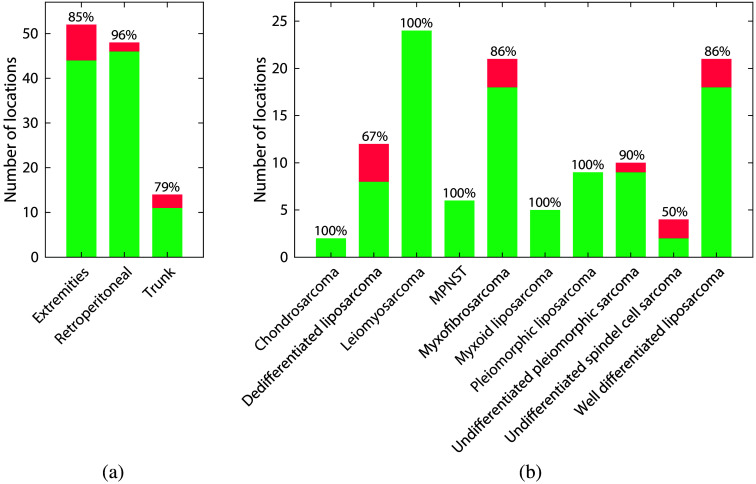
Number of correctly and incorrectly classified measurement locations for patients with (a) a specific tumor location and (b) histological subtype, with corresponding classification accuracies. These results are based on the multiclass classifier, including WDLSs. MPNST, malignant peripheral nerve sheath tumor.

To investigate the influence of different sarcoma locations (extremities, retroperitoneum, and trunk) and histological subtypes on the model performance, the tissue classification accuracy was calculated for each subgroup separately using the best performing classifier (multiclass) including WDLS. Figure 7 shows the number of correctly and incorrectly classified measurement locations for each subgroup, with corresponding accuracies. Good accuracy values were obtained for almost all sarcoma locations and subtypes. When looking at the locations, the highest accuracy was achieved for retroperitoneal tumors (96%) and the lowest with sarcomas located in the trunk (79%). Looking at different histological subtypes, we were able to correctly detect (accuracy: 100%) all chondrosarcoma, leiomyosarcoma, malignant peripheral nerve sheath tumors, myxoid liposarcoma, and pleiomorphic liposarcoma. Classifying dedifferentiated liposarcoma and undifferentiated spindle cell sarcoma were the most challenging ones.

### 3.4 Classification Performance by Neoadjuvant Therapy Status

**Fig. 8:**
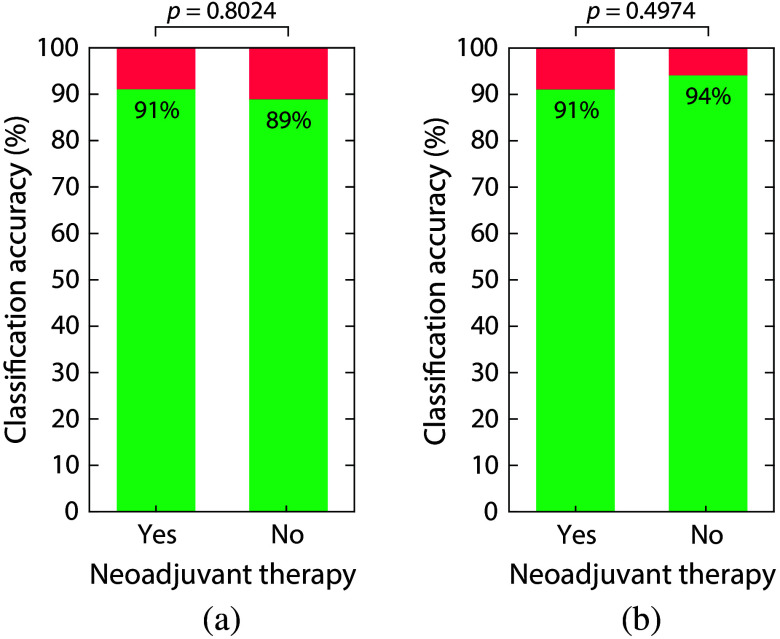
Classification accuracy for patients who received neoadjuvant therapy and patients who did not receive any neoadjuvant therapy. These results are based on the multiclass classifier, including (a) WDLSs and (b) excluding WDLSs.

To evaluate the impact of neoadjuvant therapy on the model performance, we investigated whether there are significant differences in tissue classification accuracy between patients who received neoadjuvant therapy and those who did not, using the best performing classifier (multiclass) and considering both the inclusion and exclusion of WDLS. Figure 8 shows the classification accuracy for both patient groups. The results show comparable accuracies between the two groups. When including WDLS in the analysis, the classification accuracies of 91% and 89% were achieved for patients who did and did not receive neoadjuvant therapy, respectively. Statistical analyses revealed no significant difference between the two groups (p=0.8204). When excluding WDLS, classification accuracies of 91% and 94% were achieved for patients who did and did not receive neoadjuvant therapy, respectively. Once again, statistical analyses revealed no significant difference between the two groups (p=0.4974). These findings further emphasize the robustness of the classification method across patient groups with different treatment histories.

## 4 Discussion

In this study, we investigated the potential of DRS for surgical margin assessment during STS surgery. To this end, we have collected a DRS dataset by performing measurements on both malignant and healthy locations on sarcoma specimens. Subsequently, we have developed a machine-learning model for tissue discrimination. To the best of our knowledge, this is the first study in which the DRS technique has been used for STSs. Our results demonstrated that DRS can distinguish between healthy and sarcoma tissues with good sensitivity and specificity, including different subtypes of sarcomas.

When looking at the mean DRS spectrum for different healthy and sarcoma tissues [Fig. 2(a)], it can be observed that the spectrum of sarcoma locations (excluding WDLS) differs from the spectra of healthy tissue locations in multiple wavelength regions. On the other hand, WDLS shares stronger spectral similarities to healthy fat tissue than to other sarcoma subtypes (including dedifferentiated liposarcoma). This is comparable to previous research in the literature into the correlation between lipid content and histological grade in liposarcomas.^37^ Nevertheless, t-SNE analysis seemed to reveal a subcluster of WDLS within the cluster of fat locations [Fig. 2(b)], indicating that it could still be possible to distinguish WDLS from fat tissue.

After training a tissue classification model on our acquired DRS dataset, tumor tissue could be classified with an accuracy of 0.93, sensitivity of 0.94, specificity of 0.93, and MCC of 0.86 when excluding WDLS. Including the WDLS class during classification training affects the classification performance, with a lower sensitivity of 0.88. However, lower DRS performance in the differentiation between WDLS and fat tissue is less critical from a clinical point of view since these sarcomas have a low recurrence rate and an excellent prognosis when located in the extremities.^38^ However, when present in the retroperitoneum local recurrence is seen more often and can eventually lead to mortality in these patients. Therefore, it might still be of added value to provide guidance to the surgeon for these cases as well. When taking WDLS into account as a separate class, the model was able to discriminate between fat and WDLS with reasonable accuracy. The confusion matrix in Fig. 3 shows that 75% of the WDLS locations were classified correctly, whereas 25% of the locations were misclassified as fat. This accuracy might even increase with a larger sample size and a better balance between these classes.

For all analyses, the results of a binary classification approach that distinguishes tumor tissue from all healthy tissues were compared to the results of a multiclass approach that differentiates between all different tissue types. Tables 3 and 4 showed that the highest tumor classification performance was achieved when a multiclass approach was used, both including and excluding WDLS. Combining fat, muscle, and skin tissue together in one healthy class resulted in slightly more false negative tumor predictions than when these healthy classes were treated separately, as can be seen in the confusion matrices in Figs. 3 and 5. However, it is worth noting that the binary classification results benefit from a more balanced class distribution compared to the multiclass approach, enhancing the reliability and robustness of these findings. Another observation in these confusion matrices is that muscle, skin, and WDLS are the tissue types that are most difficult to discriminate, with slightly lower sensitivity and specificity values. However, these groups are also the tissue types with the smallest number of samples per class, compared to fat and tumor classes. For these groups, it may be beneficial to acquire more data samples and examine if the classification performance for these tissue types can be improved. When specifically focusing on all misclassified locations, it was observed that it mainly concerned the same locations across all classification approaches. In addition, these locations also appeared to be in the wrong cluster in the t-SNE analysis [Fig. 2(b)]. There might be a chance that these locations are actually mislabeled in the original dataset, for example, due to a small displacement between the locations of the DRS measurement and the corresponding US image interpreted by the expert.

The developed tumor classification model showed a robust and consistent performance over all different histological subtypes and tumor locations that were included in this study, see Fig. 7. For retroperitoneal sarcomas, in which positive resection margin rates of around 45% are reported,^6^ an excellent classification accuracy of 96% was achieved. For myxofibrosarcomas, which are known for their high local recurrence rate of ∼20% to 30%,^17^^–^^20^ a promising accuracy of 86% was achieved as well. Detecting dedifferentiated liposarcomas, which originated from fatty cells but differentiated into another type, are challenging. Undifferentiated spindle cell sarcoma showed the lowest performance, but with only four examples of this class, these results should be taken with caution. For some histological subtypes and locations, classification performance may benefit from an increased number of samples. Furthermore, the developed tumor classification model showed no significant differences in performance between patients who received neoadjuvant therapy and those who did not, confirming previous conclusions in the literature^39^ and further emphasizing the robustness of the classification method across different patient groups.

A few studies can be found in the literature in which alternative optical techniques have been explored for tissue discrimination in STS specimens; see Table 5. Nguyen et al. used near-infrared autofluorescence for tissue classification, achieving an accuracy of 56% for sarcoma tissue.^22^ When excluding WDLS, the accuracy increased to 88%. In a subsequent study, Nguyen et al. explored Raman spectroscopy for sarcoma differentiation, which resulted initially in a sensitivity of 60% and specificity of 92%.^23^ When excluding WDLS, these metrics increased to 89% and 96%, respectively. Li et al. also classified STS specimens with Raman spectroscopy using both a quantitative method and a machine learning method, which resulted in a classification accuracy of 85%.^24^ Larson et al. used mid-infrared spectroscopy to show differences in absorption between sarcoma tissue and healthy tissue for laser ablation of sarcoma, but no classification was performed.^25^ Finally, Gong et al. implemented intraoperative margin assessment based on ICG in patients with STSs.^26^ The ICG margins matched the permanent pathology margins in 56% of the patients. Larger sample sizes are needed to better quantify optimal dosage, timing, and the effect of histological subtypes. In conclusion, with DRS we achieved a better performance than previously achieved with other optical techniques, especially when the WDLS subtype is included. Previous studies have repeatedly excluded WDLS during part of the analysis, resulting in increased sarcoma classification accuracies, similarly as showed in this study. However, in this study, we also examined the distinctiveness between WDLSs and healthy fat tissue specifically and showed promising results using DRS despite the small number of examples. Furthermore, from a data analysis point of view, skin has also been taken into account as an additional healthy class in this study, which has not been done before.

**Table 5:** Overview of previous studies in literature in which optical techniques were used for tissue discrimination in STSs.

Study	Optical technique	WDLS	Accuracy	Sensitivity	Specificity
Nguyen et al.^22^	NIR autofluorescence	Incl.	56%	—	—
		Excl.	88%	—	—
Nguyen et al.^23^	Raman spectroscopy	Incl.	—	60%	92%
		Excl.	—	89%	96%
Li et al.^24^	Raman spectroscopy	—	85%	—	—
Larson et al.^25^	MIR spectroscopy	—	N/A (only qualitative absorption properties)		
Gong et al.^26^	ICG	—	56%	22%	89%
Current study	DRS	Incl.	90%	88%	93%
		Excl.	93%	94%	93%

Some advantages and limitations of DRS compared to these alternative optical techniques are worth discussing. Raman spectroscopy presents the advantage of being a label-free technique without the need for contrast agents and provides detailed cellular-level information. However, it does require relatively complex instrumentation and data analysis and is limited by its shallow measurement volume and penetration depth, restricting its application to surface analysis. Fluorescence and ICG offer real-time imaging of larger surface areas, but require the administration of contrast agents and may experience limited penetration depths. On the other hand, DRS does not require contrast agents, is relatively easy to use, and provides a larger measurement volume compared to Raman, making it well-suited for examining a resection margin in real-time and identifying subsurface tumors. Moreover, the tissue discrimination performance of DRS for STS that has been achieved in this study surpasses the previously reported performances of alternative techniques in the literature. However, it is essential to acknowledge that DRS is a point-measurement technique, with limited resolution compared to Raman, and requires direct contact with the tissue. In addition, the visible part of DRS spectra can be affected by blood in *in-vivo* settings. For this reason, we have used a broad wavelength range extending up to 1600 nm, as the near-infrared region is not affected. Despite the aforementioned limitations, the practical advantages and superior tissue discrimination performance of DRS make it a promising solution for oncological surgical applications.

DRS has shown to be a promising technique for intraoperative guidance and resection margin assessment during sarcoma surgery. However, there are some points that can be addressed in future research before translation into clinical practice. First, as discussed before, the dataset used in this study had a relatively small number of samples, especially for some tissue types and histological subtypes. Future studies should aim to increase the dataset with a balanced number of samples per subtype and class, which may improve the classification performance for tissue types that were not well-represented in this study, such as muscle, skin, and WDLS. While our study employed a cross-validation approach due to the limited sample size per class, in which we took measures to reduce potential patient-specific biases through spatial separation of measurements within each patient, it is important to acknowledge the inherent limitation of this method. Future studies incorporating patient-wise cross-validation will be essential to enhance the robustness and applicability of our methodology in new clinical scenarios. Second, it would be interesting to investigate the discrimination of sarcoma from additional tissue types that surgeons would benefit from, such as blood vessels or other critical structures where sarcoma can grow close to. Third, future research should focus on the validation with histopathology results. Although the histopathology process is known to cause significant tissue deformation^40^ and the annotated ultrasound images were acquired under exactly the same circumstances as the DRS spectra, this is currently the gold standard for resection margin assessment. This will also enhance our understanding of the capabilities and limitations of DRS in critical margin or border areas, which often pose the greatest clinical challenge. The final step would be to perform an *in-vivo* study to evaluate the practical feasibility and validate the performance of DRS in the surgical setting. This technique could then even be integrated directly into surgical tools.

## 5 Conclusion

Accurately distinguishing tumor tissue from normal tissue is crucial to achieve complete resections during STS surgery while preserving critical structures. Incomplete excisions can result in local recurrences and decreased survival rates, thereby requiring subsequent surgeries or additional therapies for the patient. Noninvasive DRS technology could be used in the operating room to evaluate tissue locations in real-time. Our results showed that tumor tissue can be distinguished from different types of healthy tissue with high sensitivity and specificity using DRS, even when including WDLSs. This may improve the accuracy of tumor border detection and help surgeons achieve a better surgical outcome and improve patient prognosis.

## Data Availability

Data underlying the results presented in this paper are not publicly available at this time but may be obtained from the authors upon reasonable request.
